# Synchronized Laparoscopic Bilateral Adrenalectomy for Pheochromocytoma in Multiple Endocrine Neoplasia Syndrome: A Case Report

**DOI:** 10.15586/jkcvhl.v9i3.239

**Published:** 2022-09-02

**Authors:** Ali Eslahi, Shahryar Zeighami, Faisal Ahmed, Seyed Hossein Hosseini, Bahareh Ebrahimi, Mohammad Hossein Anbardar

**Affiliations:** 1 Department of Urology, School of Medicine, Shiraz University of Medical Sciences, Shiraz, Iran;; 2 Shiraz Geriatric Research Center, Shiraz University of Medical Sciences, Shiraz, Iran;; 3 Urology Research Center, Al-Thora General Hospital, Department of Urology, School of Medicine, Ibb University of Medical Sciences, Ibb, Yemen;; 4 Department of Pathology, School of Medicine, Shiraz University of Medical Sciences, Shiraz, Iran

**Keywords:** adrenalectomy, case report, laparoscopic, pheochromocytoma

## Abstract

Pheochromocytomas are tumors producing catecholamines that arise from chromaffin cells in the adrenal medulla. They are usually benign in multiple endocrine neoplasia type 2 (MEN2) syndrome, but they tend to present bilaterally in 50–80% of the patients. Few researchers have reported success with simultaneous laparoscopic bilateral adrenalectomy. Hence, we report a 48-year-old woman who presented with a panic attack, headache, and abdominal discomfort that had started 10 years ago. The computed tomography (CT) scan showed a large bilateral cystic lesion in both adrenal glands in favor of pheochromocytomas (30 × 22 mm and 18 × 15 mm on the right side and 40 × 33 mm and 35 × 28 mm on the left side). The patient underwent bilateral laparoscopic adrenalectomy without intraoperative or postoperative complications. The total blood loss was 50 cc, and the operative time was 4 h. The histopathology of the specimen revealed pheochromocytomas of adrenal masses. In conclusion, our case demonstrates that synchronized laparoscopic bilateral adrenalectomy can be a safe and feasible treatment option for pheochromocytomas in MEN2 patients.

## Introduction

Pheochromocytoma is a neoplasm of the adrenal glands originating from the adrenal medulla chromaffin cells, producing catecholamine (1). They are benign tumors which presented in 40–50% of type 2 multiple endocrine neoplasia (MEN2) patients aged between 30 and 40 years; also, they presented bilaterally in more than half of the cases (2). Although the advantages of unilateral laparoscopic adrenalectomy have been well documented, less experience with synchronized bilateral laparoscopic adrenalectomy has been reported in the literature (3). The surgical approaches would be determined based on the tumor size, patient’s body mass index (BMI), and the possibility of malignancy (4).

Yadav et al. described their success in treating two pheochromocytoma patients with MEN2 with simultaneous bilateral laparoscopic adrenalectomy; each surgery lasted 5 h with minimal blood loss and uneventful perioperative recovery (3).

Few researchers have reported success with laparoscopic bilateral adrenalectomy in the same operation (3, 5, 6). Hence, we present a case of simultaneous bilateral laparoscopic adrenalectomy for bilateral pheochromocytomas in a 48-year-old woman with MEN 2 syndrome focusing on surgery procedure, complications, and outcome.

## Case Report

A 48-year-old woman, a known case of diabetes mellitus who was on oral hypoglycemic medications, was referred by an endocrinologist to our urology department in September 2021 due to a history of recurrent episodes of a panic attack, headaches, and abdominal discomfort which had started 10 years ago. Recently, the patient’s son had a cerebrovascular accident that was found to have occurred secondary to pheochromocytoma. Subsequently, an endocrinologist investigated the woman for pheochromocytoma and diagnosed her with features of MEN 2. The patient was administered phenoxybenzamine 10 mg twice daily and propranolol 10 mg twice daily for blood pressure control.

The patients’ vital signs were as follows: the pulse rate: 72 beats per minute, respiratory rate: 30 per min, blood pressure: 170/90 mm Hg, and oral temperature: 37.3°C. The patient was morbidly obese with a BMI of 35 kg/m^2^, and bilateral prominent neck mass was detected in favor of enlarged thyroid glands. Another physical examination was otherwise unremarkable.

The white blood cell count was 7.3 × 10^3^/mL, hemoglobin: 11.4 g/dl, 24-h urinary excretion metanephrines: 5481 pg/day (normal: less than 350), and normetanephrine: 7732 pg/day (normal: less than 600). Thyroid function test revealed tri-iodothyronine (T3): 0.83 (normal 0.89–2.44 nmol/L), thyroxine (T4): 9.13 (normal 4.87–11.72 µg/dl), thyroid-stimulating hormone (TSH): 0.27 (normal 0.27–5.95 µIU/mL), calcitonin: 8076 (normal up to 50 pg/mL), parathyroid hormone: 71.50 (normal: 10–65 pg/ml), and fasting blood sugar (FBS): 169 mg/dl.

Thyroid ultrasonography (US) showed a bilateral enlarged heterogeneous nodule with numerous internal calcifications (left side nodule 34 × 27 mm, right side 44 × 24 mm) without cervical lymphadenopathies. The patient underwent fine-needle aspiration (FNA), and the pathology showed medullary thyroid carcinoma for thyroid mass.

**Figure 1:**
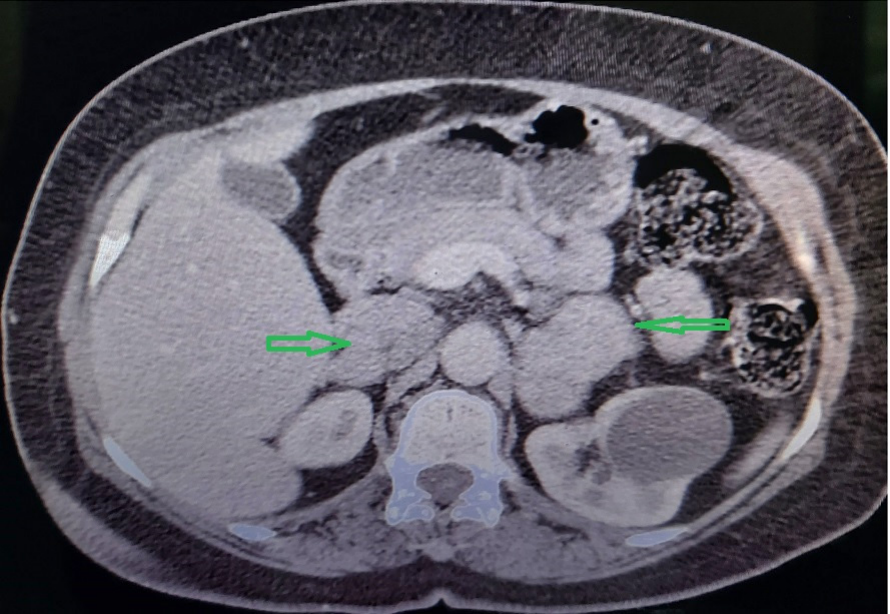
Abdominal computed tomography image showing the bilateral adrenal masses (arrows).

Contrast-enhanced abdominal computed tomography (CT) scan showed two soft tissue heterogeneous enhancement masses measuring 30 × 22 mm and 18 × 15 mm in the right adrenal gland and two soft tissue heterogeneous enhancement masses measuring 40 × 33 mm and 35 × 28 mm in the left adrenal gland (Figure 1).

**Figure 2:**
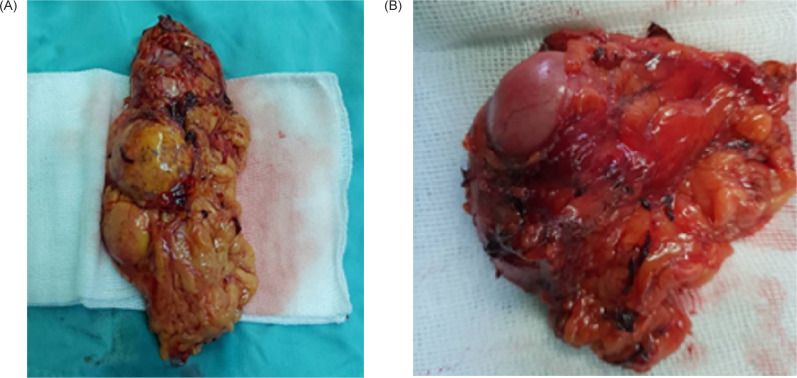
Postoperative photo of the masses. (A) Right adrenal gland. (B)Left adrenal gland).

Under general anesthesia, in the left upright position, the first four ports were inserted in the left upper, left lower, left lateral, and subcostal regions in the abdomen. Then, the adhesions were released, and the mass was explored and excised. Due to the size of the mass (8 cm), it was evacuated from the abdominal cavity via a small midclavicular port incision. Hemostasis was achieved, and a Hemovac drain was inserted. The fascia and skin were closed with Vicryl 1 and Nylon 3-0 sutures, respectively, and dressing was applied. Then, the position was changed to the right upright position. Five ports were inserted in the upper, lateral, subcostal, xyphoid, and lower regions of the right abdomen. A similar procedure was repeated, with a 6-cm mass removed via a flank incision (Figure 2). The total operative time was 4 h, and blood transfusion was not required as the total blood loss was 50 cc.

**Figure 3:**
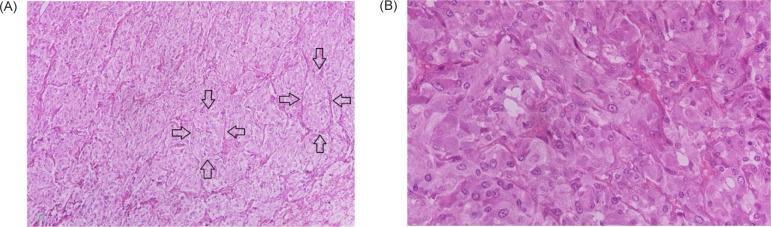
Microscopic section of adrenal mass shows: (A) Zellballen nested pattern (arrow) outlined with sustentacular cells (Hematoxylin and Eosin, 100×). (B) Polygonal tumoral cells with abundant granular cytoplasm and prominent nucleoli (Hematoxylin and Eosin, 400×).

After recovery, the patient was transferred to the urology ward and discharged without complication 3 days later. Histopathological findings showed a nested and solid pattern outlined with sustentacular cells. The tumoral cells were large and polygonal with abundant fine granular red-purple cytoplasm. The nuclei showed vesicular with variation in size and prominent nucleoli. There was no atypia, necrosis, or mitosis. The diagnosis of pheochromocytoma was confirmed morphologically (Figure 3). Postoperatively, the replacement corticosteroid therapy with hydrocortisone and later with fludrocortisone was initiated with gradual dose titration. The patient remained clinically stable in the follow-up period with a significant reduction in urinary metanephrines levels.

The hypertension crisis and the panic attack had resolved at 3-months follow-ups, and the patient was referred to a surgeon for thyroid medullary carcinoma. For that, the patient underwent a total thyroidectomy with bilateral neck dissection. Within 12 months of follow-up, the patient’s condition was improved with no future pain attacks.

## Discussion

The prevalence of bilateral pheochromocytoma mass ranges from 35 to 80%.

It seems that bilateral pheochromocytomas are caused by codon 634 and 618 mutations (2). The significant elevation of the fractionated metanephrines plasma, urine, or both in MEN 2 patients indicates the investigation of pheochromocytoma (2). Our patients’ urine metanephrines levels were significantly elevated, indicating further pheochromocytoma investigation.

Patients with pheochromocytomas typically present with various manifestations regarding adrenergic overstimulation. Sustained or paroxysmal hypertension, panic attacks, excessive sweating, and headaches indicate the diagnosis of pheochromocytomas with a specificity of 94% and a sensitivity of 91% (2, 6). Although our patient reported the referred symptoms for nearly 10 years, the diagnosis was delayed, most likely due to its paroxysmal nature.

CT scans can locate the adrenal masses bigger than 10 mm in diameter with a greater than 95% sensitivity and extra-adrenal masses bigger than 20 mm in diameter (7). The unenhanced attenuation value in Hounsfield units (HU) is used to distinguish a pheochromocytoma from a lipid-rich adenoma because attenuation in pheochromocytomas is always greater than 10 HU. If the average unenhanced attenuation value of a lesion exceeds 10 HU, histogram analysis of the unenhanced CT image can be performed; if there are 10% or more negative pixels in the histogram, an adenoma can be confirmed. In addition, in unenhanced images, pheochromocytomas had significantly higher mean grey-level intensity, entropy, and mean of positive pixels but lower skewness and kurtosis than lipid-poor adenomas (8).

The CT scan effectively showed both masses in our patient, and there was no detectable metastasis.

In MEN 2 patients, pheochromocytomas are typically treated first, while thyroid medullary carcinoma can be performed after the pheochromocytoma mass is removed to avoid intraoperative catecholamine release (3, 9). Our patient was under laparoscopic bilateral adrenalectomy and then referred to a thyroid medullary carcinoma surgery.

Blockage of both alpha-adrenergic receptor and beta-adrenergic receptor is strongly advised in the prehospital setting, between 7 and 14 days, to allow for adequate control of hypertension crises and heart rate control; such a protocol was done in our patient (6).

Although unilateral laparoscopic adrenalectomy is the ideal treatment for pheochromocytoma, there is limited experience with synchronized bilateral laparoscopic adrenalectomy (3). Furthermore, open bilateral adrenalectomy was associated with significant surgical complications such as bleeding, pancreatic fistula, incisional hernia, and delayed wound healing, while synchronized bilateral laparoscopic adrenalectomy is possible with a lower complication rate and fewer adrenal tumor manipulations (4, 10). We performed the synchronized bilateral laparoscopic surgery without complications in the operation and the postoperative period. The total intraoperative blooding was less than 50 cc, and the process lasted less than 4 h. Yadav et al. reported bilateral adrenalectomy in two MEN2 patients; the duration of the operation was 5 h, and intraoperative blooding was about 300 cc in each patient (3).

The synchronous laparoscopic bilateral adrenalectomy approaches have been described as anterior transperitoneal, lateral transperitoneal, retroperitoneal, and posterior retroperitoneal approaches (11). We used a lateral transperitoneal laparoscopic approach because most surgeons are more familiar with the anatomy and operating view. Similarly, Liao et al. performed all laparoscopic bilateral adrenalectomy with the lateral transperitoneal approach. The authors mentioned that this approach was associated with better visualization of the right adrenal, and the tumor could be accessed more efficiently during the initial positioning (12).

The main benefits of the laparoscopic technique are short hospital stay, low complication rate, and quick recovery. Compared to the posterior retroperitoneal approach, more time for changing the patient’s position, longer duration of surgery, prolonged hospital stays, and more postoperative pain are all disadvantages of the lateral approach (13). In contrast, it seems that there is no difference or superiority between the two approaches in the high BMI patients due to intricate dissection of the perirenal fat; similar results are reported by Kozłowski et al. and Chai et al. (11, 14). In addition, Conzo et al. recently stated that the lateral approach might be deemed a safe and effective technique even for pheochromocytomas larger than 6 cm in diameter (15). Our patient had a high BMI, and we think the lateral approach is preferable for both the surgeon and the patient.

Steroid replacement should start after surgery (hydrocortisone and fludrocortisone) with progressive dose titration (6). The same protocol was implemented in our patient.

## Conclusion

Our case demonstrates that synchronized laparoscopic bilateral adrenalectomy can be a safe and feasible treatment option for pheochromocytomas in MEN2 patients with low complications.

## Consent

Written informed consent was obtained as per institutional guidelines.
